# Reply to Comment on “The UK consensus position on the treatment of pancreatic cancer during the COVID-19 pandemic”

**DOI:** 10.1038/s41416-020-01133-8

**Published:** 2020-11-03

**Authors:** Christopher M. Jones, Ganesh Radhakrishna, Katharine Aitken, John Bridgewater, Pippa Corrie, Martin Eatock, Rebecca Goody, Paula Ghaneh, James Good, Derek Grose, Daniel Holyoake, Arabella Hunt, Nigel B. Jamieson, Daniel H. Palmer, Zahir Soonawalla, Juan W. Valle, Maria A. Hawkins, Somnath Mukherjee

**Affiliations:** 1 Faculty of Biological Sciences, University of Leeds, Leeds, UK; 2 Radiotherapy Research Group, Faculty of Medicsine & Health, University of Leeds, Leeds, UK; 3 Leeds Cancer Centre, The Leeds Teaching Hospitals NHS Trust, Leeds, UK; 4 The Christie NHS Foundation Trust, Manchester, UK; 5 The Royal Marsden Hospital, The Royal Marsden NHS Foundation Trust, London, UK; 6 The Institute of Cancer Research, London, UK; 7 University College London Cancer Institute, London, UK; 8 Department of Oncology, Cambridge University Hospitals NHS Foundation Trust, Cambridge, UK; 9 The Northern Ireland Cancer Centre, Belfast, UK; 10 The Royal Liverpool University Hospital, Liverpool, UK; 11 University Hospitals Birmingham NHS Foundation Trust, Birmingham, UK; 12 Beatson West of Scotland Cancer Centre, Glasgow, UK; 13 Norfolk & Norwich University Hospitals NHS Foundation Trust, Norwich, UK; 14 Wolfson Wohl Cancer Research Centre, University of Glasgow, Glasgow, UK; 15 The Clatterbridge Cancer Centre NHS Foundation Trust, Liverpool, UK; 16 Liverpool Experimental Cancer Medicine Centre, University of Liverpool, Liverpool, UK; 17 Oxford University Hospitals NHS Foundation Trust, Oxford, UK; 18 Division of Cancer Sciences, University of Manchester, Manchester, UK; 19 Department of Medical Physics & Biomedical Engineering, University College London, London, UK; 20 CRUK/MRC Oxford Institute for Radiation Oncology, University of Oxford, Oxford, UK

**Keywords:** Pancreatic cancer, Pancreatic cancer

We read with interest Arshad et al.’s thoughts on our guidance, which was made available to and revised in response to feedback from clinicians from the outset of the COVID-19 pandemic.^1,2^ We note that the authors have not commented on any specific aspect of the article’s content; instead proffering their appraisal of the authors, coupled with a call for business as usual alongside an inference that amidst an unprecedented pivot in NHS care towards patients with COVID-19 at a time when up to a fifth of medical staff were off work, such guidance was redundant.^3–7^ The in-excess of 2200 downloads of, and over 400 visits to, the treatment protocols and webpages respectively associated with this guidance suggest such conjecture to be erroneous, but we fully dissect their multiple assumptions in turn here.^8^

Firstly, Arshad et al. highlight that though the authorship is geographically broad, it does not include representation from colleagues typically involved in the diagnosis or palliation of patients with pancreatic cancer. The article is explicit in defining its scope as extending to areas in which the safe treatment of patients with pancreatic cancer during the first or any subsequent peaks in COVID-19 incidence might be impeded. Additional domains, such as those relating to diagnosis and for which we would entirely agree additional representation would be required, have been outlined in detail elsewhere.^9–13^ Arshad et al. may be reassured that there are no fewer than seven references to guidance provided by other professional groups within the paper to reflect this.

Secondly, Arshad et al. seek to define the extent to which the wider pancreatic cancer community have been consulted with respect to the guidance. As is detailed within the manuscript, feedback was provided by patient and public representatives via the charity, Pancreatic Cancer UK. A broader initiative, run as part of the patient-facing Pancreatic Cancer during COVID-19 (PCC) network to which these authors have contributed and supported, saw the guidance discussed and opened for feedback within at least two of twelve COVID-19-focussed webinars that have been attended by a majority of major surgical and non-surgical UK pancreatic cancer treatment centres. Further substantial representation was achieved at pace at the height of an evolving pandemic through the establishment of a dedicated website (www.uppergicancer.com), via participation in an online forum convened by the Royal College of Radiologists (RCR) and via the initiation of a peer-support mechanism for clinicians seeking advice on treatment decisions for patients with pancreatic cancer. Quite in contrast to Arshad et al.’s speculation, the culminating manuscript was peer-reviewed, much like any other, and is published online to allow for critique such as that we are responding to here.

Thirdly, Arshad and colleagues query the methodology used in the development of the guidance and the potential for a ‘predominance of clinical oncologists’ in the authorship to have resulted in ‘excess detail on the radiation oncology management in the paper’. Less than 15% (316 words) of the paper’s 2390 words focus on the radiation-based management of pancreatic cancer, which is hardly a ‘singular feature’. Further, and as Arshad and colleagues are presumably aware, there is no specialty of radiation oncology in the UK and as such 15 of the 18 authors are trained and have experience of the systemic therapies to which the bulk of the article relates. We are then unsure of the accuracy of the metrics on which Arshad and his four colleagues have relied. In fact, where radiation is discussed it is almost universally with respect to the use of hypo-fractionated regimens, a treatment approach that has been widely supported across the world during the pandemic.^14,15^

Fourthly, and turning to its formation, we agree with Arshad et al. that constrained by a need to urgently provide clinicians with guidance, we were not able to fully align our methodology with gold standard practice, and that it is possible that another eighteen authors may have proposed alternative guidance. However, we are not aware of—and we note that Arshad and colleagues have not provided—such an alternative. Further, whilst we agree and indeed highlight in the manuscript that thankfully ‘many sites had very little disruption… even at the height of the pandemic’, the obvious corollary is that many did face significant disruption.

Thus, we agree with Arshad et al. that as the first pandemic peak subsides there is now ‘no reason why the management of pancreas cancer should be different’, excepting the testing and distancing measures outlined by Arshad and colleagues that make this possible. However, the utility of our guidance for clinicians who grappled with maintaining pancreatic cancer treatments in the absence of these very measures and in the midst of swingeing restrictions to NHS services is made clear here, as are our significant efforts to engage at pace with the pancreatic cancer community.

## Data Availability

Not applicable.
